# Supplementary material for: ADAMTS4-specific MR probe to assess aortic aneurysms in vivo using synthetic peptide libraries
Source: Nat Commun. 2022 May 23;13:2867. doi: 10.1038/s41467-022-30464-8 (PMC9126943; doi:10.1038/s41467-022-30464-8)
Supplement: Supplementary file 1 — Supplementary information [file 41467_2022_30464_MOESM1_ESM.docx]

**Supplementary Information**

**ADAMTS4-specific MR-probe to assess aortic aneurysms in vivo using synthetic peptide libraries**

Jan O. Kaufmann^1,2,3°^_,_ Julia Brangsch^1,4°^, Avan Kader^1,5,6^, Jessica Saatz^7^, Dilyana B. Mangarova^1,8^, Martin Zacharias^9^, Wolfgang E. Kempf^10^, Timm Schwaar^11^, Marco Wilke^3^, Lisa C. Adams^1^, Jana Möckel^1^, Rene M. Botnar^12,13,14,15,16^, Matthias Taupitz^1^, Lars Mägdefessel^10^, Heike Traub^7^, Bernd Hamm^1^, Michael G. Weller^3^, Marcus R. Makowski^1,6,12*^

°The authors contributed equally

Supplementary Figures 1-18 (p. 2-30)

Supplementary Tables 1-7 (p. 31-38)

## Supplementary Figure 1

**Supp. Figure 1: Analysis of the fluorescence scan of the five respective peptides on chip, the MALDI-TOF-MS sequencing and the SPR evaluation of the five respective peptides.**

**a**, The chip was scanned at 635 nm. Between each scan, the chip was regenerated with guanidium HCl to denature all proteins from the peptides on the resin. Four different scans were made. The first one, the prescan, contained only both staining antibodies, while the second and third were used with ADAMTS4 and ADAMTS5 to check also the selectivity of the peptides. The last one was only with BSA as a control scan for checking if the peptide resin was still in position. For evaluation of the scans, a ROI of 225 µm^2^ (9 pixels) were used and the mean fluorescence was measured. For the ratio of fluorescence between the ADAMTS4 scan and the ADAMTS5 scan, both values were subtracted with the prescan value and afterward divided. While peptide 7 and 16 showed higher selectivity for ADAMTS4, peptide 11, 12 and 15 showed slightly higher binding to ADAMTS5. Surprisingly, peptide 16 showed the highest selectivity in the screening even though it has two arginine moieties, which is normally more common for unselective binding. **b**, MALDI-TOF-MS analysis of the five highest hits in the ADAMTS4 screening. The MALDI-TOF-MS spectra showed good or very good truncation sequences for the peptides 11, 12, and 16, while for peptide 7 and 15 the sequence was not definite. Since both peptides demonstrated only a weak binding in the SPR, a misanalysis could be possible. In the table a6 all sequences of the peptide cycle were summarized. Surprisingly, peptide 16 showed the highest selectivity in the screening even though it has two arginine moieties, which is normally more common for unselective binding. **c**, After resynthesis of the five sequences with the linker part, we examined the binding constant of them to ADAMTS4 by using SPR spectroscopy. We used a captured chip immobilization of ADAMTS4 by applying the anti-His-ab to the hydrogel layer first, followed by the ADAMTS4. In the left spectrogram, the steady states of the different peptide concentrations were applied. For the peptide 11, 12, and 16, lower concentrations had to be measured, due to the high signal response. The right spectrogram is a magnification of the left one. After the measurement of all five peptides, peptide number 16 showed the highest signal at the lowest concentration. MALDI-TOF-MS: Matrix-assisted laser desorption/ionization-time of flights-mass spectrometry; nm: nanometer; HCl: hydrochloride; BSA: Bovine serum albumin; ROI: region of interest; fi: fluorescence intensity; SPR: surface plasmon resonance.

## Supplementary Figure 2

**Supp. Figure 2: SPR diagramm of the peptides and the capturing and immobilisation of anti-His-ab.**

**a,**Represents the immobilization of the anti-His-tag ab on a hydrogel gold chip. The chip was activated twice with EDC and NHS. At the second time, some air was injected unintentional, too. The antibody was injected over both channels, even though most of it was bound on the sample channel. Afterwards the chip was quenched by aminoethanol. **b,** Shows the capturing of ADAMTS4. ADAMTS4 was injected over the sample channel for 15 min. Afterwards, the diverter was open, and buffer was flushing over both channels, until the baseline was nearly stable. The amount of captured ADAMTS4 was calculated for 26% of the Rmax. **c-g** represents the SPR spectra of the 5 peptides from the screening. The peptides were used as crude products. For each peptide different concentrations were measured. Peptide 7 (**c**) and peptide 15 (**f**) did show the weakest signal. Peptide 11 (**d**) and 12 (**e**) showed similar signals, while peptide 16 (**g**) showed the highest signal.

SPR: surface plasmon resonance; EDC: 1-Ethyl-3-(3-dimethylaminopropyl)carbodiimide; NHS: *N-*hydroxysuccinimid; R_max_: maximal response.

## Supplementary Figure 3

**Supp. Figure 3: Binding analysis of the ADAMTS4-binding peptide with ADAMTS1 and ADAMTS5 and an alanine scan for the peptide cycle.**

**a,**The table shows all data of all MST measurements. All measurements were performed with 10% LED power, high MST power and at 25 °C. The ligand dependent fluorescence change was used for determining the binding. The values represent the data from the MO Affinity analysis software. **b,**In this MST experiment, ADAMTS1 and ADAMTS5 were used in the same way like ADAMTS4 with the Cy5 labeled peptide. The concentrations of the peptide were kept constant (3 nM in the measurement), while the proteins were diluted in the same manner as for ADAMTS4. A beginning binding was detected, but the plateau was not reached (n=3 biologically independent measurements for each peptide; the data are presented as mean values ± SD). **c,**We performed an alanine scan for the cyclic peptide structure, where each amino acid was replaced respectively with alanine. The Cy5-labeled peptides (3 nM in the measurement) were kept constant, while ADAMTS4 was diluted in the same manner for each peptide (n=3 biologically independent measurements for each peptide; the data are presented as mean values ± SD). While the exchange of Gly and Ser decrease the binding, the binding is completely loss by the exchange of each arginine. MST: microscale thermophoresis; nM: nanomolar; His: L-histidine; LED: light-emitting diode; n/a: not available; K_D_: binding constant.

## Supplementary Figure 4

**Supp. Figure 4: MST capillary scans and MST data for the three peptides without ligand dependent fluorescence change.**

Represents the 8 MST capillary scans of all three biological replicates. While for the ADAMTS4-specific probe against ADAMTS4 (**a**), ADAMTS1 (**c**) and ADAMTS5 (**d**) as well as for the ADAMTS4-specific probe-Gly (**e**) and –Ser (**h**) a ligand dependent fluorescence change occurs, for the negative control probe (**b**), ADAMTS4-specific probe-Arg1 (**f**) and –Arg2 (**g**) no fluorescence change was detectable. For this three (**i**, **j**, **k**), we checked also the MST points at 2.5 seconds and no binding occurs neither.

MST: microscale thermophoresis.

## Supplementary Figure 5

**Supp. Figure 5: Docking with mouse ADAMTS4.**

Simulation snapshot from the simulation (~25 ns) of the mouse ADAMTS4 in complex with peptide binder. Mouse ADAMTS4 in orange cartoon, Glu397,398 as sticks, peptide as stick model including hydrogens.

## Supplementary Figure 6

**Supp. Figure 6: Simulation repetition and mutation of ADAMTS4.**

**a,**Root-mean-square deviation (RMSD of non-hydrogen atoms) vs. simulation time for three separate simulations of ADAMTS4 in complex with peptide binder (A-C). Simulations were started with different initial velocity distributions. The RMSD of the complete protein vs. simulation time is indicated in black, the RMSD of the peptide binder with respect to the start placement (after best superposition of the protein on the start structure) is shown in red. (D) same simulation for the mouse ADAMTS4 in complex with the peptide binder (same initial position as in human ADAMTS4 cases). (E) same as in (A) but with the in silico human ADAMTS4 Glu401Gln, Glu402Gln mutation. Note, the RMSD scale is different in (E). **b,**Simulation snapshots of the in silico double mutation (Glu401Gln, Glu402Gln) at early simulation stage (A, ~2 ns) and upon complete dissociation of the peptide (B, ~25 ns). Human ADAMTS4 in orange cartoon, mutated residues as sticks, peptide as stick model including hydrogens.

## Supplementary Figure 7

**Supp. Figure 7: Cytotoxicity test on human aortic endothelial cells (HAEC) and mouse aortic endothelial cells (MAEC).**

**a,** MTT-assay with (left) HAEC and MAEC (right) on different time points (3 h, 24 h, 48 h) and different concentrations of ADAMTS4 probe (0.3, 0.6, 0.8, 2.2, 4.4 and 8.8 µmol). The difference between the concentrations and the negative control was not significant (human: 3 h:P=0.542; 24 h=0.231; 48 h=0.183; mouse: 3 h:P=0.790; 24 h=0.265; 48 h=0.065). A significant difference was found between the positive control and the concentrations (human: 3 h:P=48.41E-10; 24 h=2.55E-11; 48 h=4.51E-05; mouse: 3 h:P=4.19E-12; 24 h=1.75E-13; 48 h=7.90E-12). pc = positive control; nc = negative control. For each concentration n=3 biologically independent measurements were performed **b,** Cytotox-One assay with (left) HAEC and MAEC (right) on different time points (3 h, 24 h, 48 h) and concentrations of ADAMTS4 probe (0.3, 0.6, 0.8, 2.2, 4.4 and 8.8 µmol). The percentage of cytotoxicity was determined. For each concentration n=4 biologically independent measurements were performed. **c,** Celltiter-Glo assay with (left) HAEC and MAEC (right) on different time points (3 h, 24 h, 48 h) and concentrations of ADAMTS4 probe (0.3, 0.6, 0.8, 2.2, 4.4 and 8.8 µmol). There was no significant difference between the concentrations and the negative control (human: 3 h:P=0.429; 24 h=0.075; 48 h=0.072; mouse: 3 h:P=0.277; 24 h=0.075; 48 h=0.074). Significant difference was found in positive control and concentrations (human: 3 h:P=1.15E-18; 24 h=1.75E-29; 48 h=4.37E-21; mouse: 3 h:P=3.20E-27; 24 h=1.75E-29; 48 h=2.55E-23). For each concentration n=6 biologically independent measurements were performed; the data are presented as mean values ± SD.

PC = positive control, NC= negative control, RLU= relative light unit.

## Supplementary Figure 8

**Supp. Figure 8: *In vitro* toxicity assay and *in vivo* biodistribution analysis of the ADAMTS4-specific probe.**

**a,** Tissue uptake and retention overtime of the ADAMTS4-specific probe were evaluated in 9 ApoE^-/-^ mice. Aortic uptake was highest after 15 min after intravenous injection. Levels of the ADAMTS4-specific probe in kidney and urine were high, whereas liver levels retained low. **b,** The blood concentration of the ADAMTS4-specific probe was determined in 9 ApoE^-/-^ mice over 120 min and indicated a rapid blood clearance.

## Supplementary Figure 9

**Supp. Figure 9: Experimental setup and timelines of the animal experiments.**

**a,** In the cross-sectional study, MR imaging was performed after two and four weeks of AngII-infusion (n=10 per group), while a sham group (n=10) was receiving saline for 28 days instead of AngII. **b,** For the longitudinal study, mice (n=12) were imaged after one week of AngII-infused with the ADAMTS4-specific probe. After four weeks, they were sacrificed, and aortas excised. **c,** The early detection study includes MR imaging sessions after 2, 4, and 10 days after AngII-infusion (n=20) or saline infusion for the sham group (n=5) with the ADAMTS4-specific probe. After 28 days, the mice were scanned with a native MRI and sacrificed.

## Supplementary Figure 10

**Supp. Figure 10: Expression of ADAMTS4 can be performed by differing cell types.**

Expression of macrophages (**a**), ADAMTS4 (**b**, **e**) and SMCs (**d**) in the same tissue slide. The overlay of ADAMTS4 with CD68 (**c**) and SMCs (**f**) demonstrate strong colocalization but shows also that ADAMTS4 is expressed by different cell types. *Ex vivo* immunofluorescence images of subsequent AAA slides following 4 weeks of Ang II infusion demonstrate the co-localization of ADAMTS4 with SMCs and CD68-positive cells. Scale bars represent 100 µm.

SMC: smooth muscle cell

## Supplementary Figure 11

**a**

**b**

**c**

**Supp. Figure 11: Structure and MALDI-ToF-MS of Peptide 7.**

**a,** Shows the structure of Peptide 7. **b,c** Shows the MALDI-ToF-MS of Peptide 7 after cleavage. The peptide was used without further purification.

## Supplementary Figure 12

**a**

**b**

**c**

**Supp. Figure 12: Structure and MALDI-ToF-MS of Peptide 11.**

**a,** Shows the structure of Peptide 11. **b,c** Shows the MALDI-ToF-MS of Peptide 11 after cleavage. The peptide was used without further purification.

## Supplementary Figure 13

**a**

**b**

**c**

**Supp. Figure 13: Structure and MALDI-ToF-MS of Peptide 12.**

**a,** Shows the structure of Peptide 12. **b,c** Shows the MALDI-ToF-MS of Peptide 12 after cleavage. The peptide was used without further purification.

## Supplementary Figure 14

**a**

**b**

**c**

**Supp. Figure 14: Structure and MALDI-ToF-MS of Peptide 15.**

**a,** Shows the structure of Peptide 15. **b,c** Shows the MALDI-ToF-MS of Peptide 15 after cleavage. The peptide was used without further purification.

## Supplementary Figure 15

**a**

**b**

**c**

**Supp. Figure 15: Structure and MALDI-ToF-MS of Peptide 16.**

**a,** Shows the structure of Peptide 16. **b,c** Shows the MALDI-ToF-MS of Peptide 16 after cleavage. The peptide was used without further purification.

## Supplementary Figure 16

**a**

**b**

**c**

**Supp. Figure 16: Complexation of Gd(III) to the ADAMTS4-specific probe.**

**a,** Shows the reaction scheme. **b** Shows the MALDI-ToF-MS of the ADAMTS4-specific probe after complexation. **c** Shows the FPLC-chromatogram of the desalting step after the complexation.

## Supplementary Figure 17

**a**

**b**

**c**

**Supp. Figure 17: Complexation of Eu(III) to the ADAMTS4-specific probe.**

**a,** Shows the reaction scheme. **b** Shows the MALDI-ToF-MS of the ADAMTS4-specific probe after complexation of europium. **c** Shows the FPLC-chromatogram of the desalting step after the complexation.

## Supplementary Figure 18

**a**

**b**

**c**

**Supp. Figure 18: Complexation of Gd(III) to the negative control probe.**

**a,** Shows the reaction scheme. **b** Shows the MALDI-ToF-MS of the negative control probe after complexation of gadolinium. **c** Shows the FPLC-chromatogram of the desalting step after the complexation.

Supplementary Tables

**Supp. Table 1: Reagents for synthesis of HMBA linker and spacer on resin**

Amount of amino acids:

|  | m [mg] | n [µmol] | Equivalents |
| --- | --- | --- | --- |
| Fmoc-L-Arg(Pbf)-OH | 1219.7 | 1880 | 4 |
| Fmoc-L-Ser(tBu)-OH | 828.1 | 1880 | 4 |
| Fmoc-TDDS-OH | 1275.2 | 2350 | 5 |
| HCTU | 778 | 1880 | 4 |
| NMM | 380.5 (413.6 µl) | 3760 | 8 |

**Supp. Table 2: Reagents for synthesis of library**

Amount of reagents:

For each coupling 20 µl NMM (182 µmol), 37.6 mg HCTU (91 µmol) and 1 ml of DMF were used additionally to the amino acids.

Arginine:

| Fmoc-L-Arg(Pbf)-OH | | Boc-L-Arg(Pbf)-OH | |
| --- | --- | --- | --- |
| 51.5 mg | 84.6 µmol | 3.1 mg | 6.4 µmol |

Asparagine:

| Fmoc-L-Asn(Trt)-OH | | Boc-L-Asn(Trt)-OH | |
| --- | --- | --- | --- |
| 31.2 mg | 84.6 µmol | 2.6 mg | 6.4 µmol |

Glutamic acid:

| Fmoc-L-Glu(tBu)-OH | | Boc- L-Glu(tBu)-OH | |
| --- | --- | --- | --- |
| 36 mg | 84.6 µmol | 1.9 mg | 6.4 µmol |

Glycine:

| Fmoc-Gly-OH | | Boc-Gly-OH | |
| --- | --- | --- | --- |
| 25.2 mg | 84.6 µmol | 1.1 mg | 6.4 µmol |

Histidine:

| Fmoc-L-His(Trt)-OH | | Boc-L-His(Trt)-OH | |
| --- | --- | --- | --- |
| 45.4 mg | 84.6 µmol | 2.6 mg | 6.4 µmol |

Phenylalanine:

| Fmoc-L-Phe-OH | | Boc-L-Phe -OH | |
| --- | --- | --- | --- |
| 33.6 mg | 84.6 µmol | 1.7 mg | 6.4 µmol |

Proline:

| Fmoc-L-Pro-OH | | Boc-L-Pro-OH | |
| --- | --- | --- | --- |
| 28.6 mg | 84.6 µmol | 1.4 mg | 6.4 µmol |

Serine:

| Fmoc-L-Ser(tBu)-OH | | Boc-L-Ser-OH | |
| --- | --- | --- | --- |
| 32.4 mg | 84.6 µmol | 1.3 mg | 6.4 µmol |

Tryptophane:

| Fmoc-L-Trp(Boc)-OH | | Boc-L-Trp-OH | |
| --- | --- | --- | --- |
| 44.5 mg | 84.6 µmol | 1.9 mg | 6.4 µmol |

Tyrosine:

| Fmoc-L-Tyr(tBu)-OH | | Boc-L-Tyr-OH | |
| --- | --- | --- | --- |
| 38.9 mg | 84.6 µmol | 1.8 mg | 6.4 µmol |

Valine:

| Fmoc-L-Val-OH | | Boc-L-Val-OH | |
| --- | --- | --- | --- |
| 28.7 mg | 84.6 µmol | 1.4 mg | 6.4 µmol |

**Supp. Table 3: Reagents for the resynthesis of the positive hits**

For the resynthesis of the five positive hits the following amounts of amino acids and reagents were used in 1 ml DMF:

|  | m [mg] | n [µmol] | equivalents |
| --- | --- | --- | --- |
| Fmoc-L-Arg(Pbf)-OH | 75.6 | 117 | 8 |
| Fmoc-L-Cys(Trt)-OH | 68.5 | 117 | 8 |
| Fmoc-L-Glu(tBu)-OH | 49.6 | 117 | 8 |
| Fmoc-Gly-OH | 34.7 | 117 | 8 |
| Fmoc-L-His(Trt)-OH | 62.6 | 117 | 8 |
| Fmoc-L-Phe-OH | 46.3 | 117 | 8 |
| Fmoc-L-Pro-OH | 39.4 | 117 | 8 |
| Fmoc-L-Ser(tBu)-OH | 51.3 | 117 | 8 |
| Fmoc-L-Tyr(tBu)-OH | 53.6 | 117 | 8 |
| Fmoc-L-Val-OH | 39.6 | 117 | 8 |
| HCTU | 48 | 117 | 8 |
| NMM | 26 µl | 234 | 16 |

**Supp. Table 4: MS-data for the resynthesized peptides**

| Peptide | m/z [M+H]^+^ | | m/z [M+Na]^+^ | | m/z [M+K]^+^ | |
| --- | --- | --- | --- | --- | --- | --- |
|  | calculated | found | calculated | found | calculated | found |
| Pep7 | 1523.76 | 1524.15 | 1545.74 | 1546.14 | 1761.71 | 1562.15 |
| Pep11 | 1679.81 | 1680.27 | 1701.8 | 1702.28 | 1717.77 | 1718.22 |
| Pep12 | 1815.83 | 1816.36 | 1837.81 | 1838.36 | 1853.79 | 1854.32 |
| Pep15 | 1757.83 | 1758.35 | 1779.82 | 1780.42 | 1795.79 |  |
| Pep16 | 1669.85 | 1670.42 | 1691.83 | 1692.37 | 1707.8 | 1708.33 |

**Supp. Table 5: MS-data for the complexation of Eu(III) or Gd(III) to the peptides**

| Peptide | m/z [M+H]^+^ | | m/z [M+Na]^+^ | | m/z [M+K]^+^ | |
| --- | --- | --- | --- | --- | --- | --- |
|  | calculated | found | calculated | found | calculated | found |
| ADAMTS4-specific probe (Gd) | 1806.64 | 1806.42 | 1828.62 | 1828.39 | 1844.6 | 1844.38 |
| ADAMTS4-specific probe (Eu) | 1801.64 | 1801.08 | 1823.62 | 1823.07 | 1839.59 |  |
| Negative control probe [Gd] | 1894.63 | 1894.76 | 1916.61 | 1916.72 | 1932.58 | 1932.68 |

The peptides were purchased from peptides&elephants GmbH (Hennigsdorf, Germany) with purity of at least 95%. Each peptide was delivered with a certification of analysis. The mass shift due to the complexation of the metal ion to DOTA was confirmed via MALDI-ToF-MS. The calculated mass [M+H]^+^ for the peptides before complexation are for the ADAMTS4-specific probe m/z: 1651.74 and for the Negative control probe m/z: 1739.73.

**Supp. Table 6:** **Experimental parameters of the iCAP Qc**

| **Parameter** | **Value** |
| --- | --- |
| Power [W] | 1550 |
| Nebulizer gas flow rate [L min^−1^] | 1.08 |
| Aux gas flow rate [L min^−1^] | 0.65 |
| Cool gas flow rate [L min^−1^] | 14 |
| Sample flow rate [mL min^−1^] | 0.40 |
| Dwell time [ms] | 0.05 |
| Isotopes monitored | ^157^Gd,^158^Gd,^165^Ho |

**Supp. Table 7: Instrumental parameters of the LA-ICP-MS system**

| **ICP-MS** | | **LA system** | |
| --- | --- | --- | --- |
| RF plasma power [W] | 1350 | Wavelength [nm] | 213 |
| Plasma gas flow (Ar) [L min^-1^] | 16 | Helium gas flow [L min^-1^] | 1 |
| Sample gas flow (Ar) [L min^-1^] | 0.560 | Laser energy [J cm^-2^] | 2.4 |
| Auxiliary gas flow (Ar) [L min^-1^] | 1.05 | Laser spot size [µm] | 30 |
| Mass resolution (*m/∆m*) | 300 (LR) | Scan speed [µm s^-1^] | 30 |
| Sample time [ms] | 2 | Repetition rate [Hz] | 20 |
| Scanning mode | Line by line | Line overlap [µm] | 15 |
| Detected isotopes | ^31^P, ^34^S, ^57^Fe, ^65^Cu, ^66^Zn, ^158^Gd, ^153^Eu, ^160^Gd |  |  |
