# Supplementary material for: ADAMTS4-specific MR probe to assess aortic aneurysms in vivo using synthetic peptide libraries
Source: Nat Commun. 2022 May 23;13:2867. doi: 10.1038/s41467-022-30464-8 (PMC9126943; doi:10.1038/s41467-022-30464-8)
Supplement: Supplementary file 2 — Reporting Summary [file 41467_2022_30464_MOESM2_ESM.pdf]

nature portfolio

Corresponding author(s): Prof. Dr. Marcus Makowski

Last updated by author(s): Apr 19, 2022

Reporting Summary

Nature Portfolio wishes to improve the reproducibility of the work that we publish. This form provides structure for consistency and transparency in reporting. For further information on Nature Portfolio policies, see our Editorial Policies and the Editorial Policy Checklist.

Statistics

For all statistical analyses, confirm that the following items are present in the figure legend, table legend, main text, or Methods section.

n/a	Confirmed	
☐	☒	The exact sample size (*n*) for each experimental group/condition, given as a discrete number and unit of measurement
☐	☒	A statement on whether measurements were taken from distinct samples or whether the same sample was measured repeatedly
☐	☒	The statistical test(s) used AND whether they are one- or two-sided<br>*Only common tests should be described solely by name; describe more complex techniques in the Methods section.*
☒	☐	A description of all covariates tested
☐	☒	A description of any assumptions or corrections, such as tests of normality and adjustment for multiple comparisons
☐	☒	A full description of the statistical parameters including central tendency (e.g. means) or other basic estimates (e.g. regression coefficient) AND variation (e.g. standard deviation) or associated estimates of uncertainty (e.g. confidence intervals)
☐	☒	For null hypothesis testing, the test statistic (e.g. *F*, *t*, *r*) with confidence intervals, effect sizes, degrees of freedom and *P* value noted<br>*Give P values as exact values whenever suitable.*
☐	☒	For Bayesian analysis, information on the choice of priors and Markov chain Monte Carlo settings
☐	☒	For hierarchical and complex designs, identification of the appropriate level for tests and full reporting of outcomes
☒	☐	Estimates of effect sizes (e.g. Cohen's *d*, Pearson's *r*), indicating how they were calculated

Our web collection on statistics for biologists contains articles on many of the points above.

Software and code

Policy information about availability of computer code

Data collection	Data were collected by instrument specific software: Microarray scanner; MST (MO.Control v2.0.3, NanoTemper, Germany); MALDI-TOF-MS; SPR (Reichert Autolink, 1.1.14-T, Reichert, USA);
Data analysis	ImageJ (1.51, National Institute of Health, USA); TraceDrawer (1.6.1, Ridgeview Instruments, Uppsala, Sweden); Scrubber2 (BioLogic Software, Canberra, Australia); Origin 2022 (OriginLab Corporation, Northampton, MA); MO.Affinity Analysis v3.0.1 (NanoTemper, Munich, Germany); ATTRACT Version 1 (http://www.attract.ph.tum.de/services/ATTRACT/ATTRACT.vdi.gz; TUM, Germany); Amber18 (http://ambermd.org/index.php, University of California, San Fancisco, USA); Keyence BZX-800 Analyzer (Keyence, Japan); ImageScope_64_v12.4.0.5043 (Leica, Germany); Excel Version 16.57, Micrsoft®, Washington, USA, OsiriX (version 7.1, OsiriX foundation, Bernex, Switzerland)

For manuscripts utilizing custom algorithms or software that are central to the research but not yet described in published literature, software must be made available to editors and reviewers. We strongly encourage code deposition in a community repository (e.g. GitHub). See the Nature Portfolio guidelines for submitting code & software for further information.

Data

Policy information about availability of data

All manuscripts must include a data availability statement. This statement should provide the following information, where applicable:

- Accession codes, unique identifiers, or web links for publicly available datasets
- A description of any restrictions on data availability
- For clinical datasets or third party data, please ensure that the statement adheres to our policy

The datasets generated during and/or analysed during the current study are available from the corresponding author on reasonable request.

Field-specific reporting

Please select the one below that is the best fit for your research. If you are not sure, read the appropriate sections before making your selection.

☒ Life sciences ☐ Behavioural & social sciences ☐ Ecological, evolutionary & environmental sciences

For a reference copy of the document with all sections, see nature.com/documents/nr-reporting-summary-flat.pdf

Life sciences study design

All studies must disclose on these points even when the disclosure is negative.

Sample size

HPLC-stability test was performd only once. Since there were no difference for the different time points for the probe, this experiment is sufficient. SPR analysis was performed only once, since it was only for classify all hits for binding or no-binding. For this classification 5 measurements were perfomred to cover the concentrational range.
MST analysis was repeated three times for each system and the confidence intervall was stated. According to Nanotemper, three independent repeation for MST are sufficient for binding determination.
For MTT analysis, CytoTox-One and Celltiter-Glo two different cell lines were used and measured with different concentrations of MRI probe at 3 different time points (3h, 24h, 48h) according to comparable studys and standard procedure.
For the animal testing, a power analysis was used for the total amount of animals per group. For the cross-sectional study, 10 mice per group were scanned in the MRI, while for longitudinal study 12 mice per group were scanned. The early detection study contained 20 mice in the beginning.
For ex vivo examination by LA-ICP-MS and ICP-MS three independent samples were used for each experiment. For the biodistribution three independent samples were used for each organ or at each time point. Sample sizes of these ex vivo experiments were limited due to the limited amount of animals/tissue and with respect to the 3R priniciples. The sample size of 3 per measurement was considered as sufficent with reference to previous studies.

Data exclusions

No data were excluded.

Replication

For MST measurement, ICP-MS and LA-ICP-MS three independent repetitions were performed and the merged data were used for the respective analysis. For the MRI in vivo analysis, 10, 12 or 20 animals were scanned for further analysis using genetically identical apolipoprotein-E knockout (ApoE-/-) (B6.129P2-ApoEtm1Unc/J) mice. Histological analyses were performed for all animals following in vivo imaging. All attempts at replication were successful. For MTT analysis, CytoTox-One and Celltiter-Glo two different cell lines were used and measured with different concentrations of MRI probe at different time points. For MTT, each concentration was repeated 3 times for each timepoint, CytoTox-One was repeated 4 times, Celltiter-Glo was repeated 6 times. All attempts at replication were successful.

Randomization

Samples/organisms were randomized into the different groups.

Blinding

Investigators were blinded to group allocation during data collection and analysis.

Reporting for specific materials, systems and methods

We require information from authors about some types of materials, experimental systems and methods used in many studies. Here, indicate whether each material, system or method listed is relevant to your study. If you are not sure if a list item applies to your research, read the appropriate section before selecting a response.

Materials & experimental systems

n/a	Involved in the study	
☐	☒	Antibodies
☐	☒	Eukaryotic cell lines
☒	☐	Palaeontology and archaeology
☐	☒	Animals and other organisms
☐	☒	Human research participants
☒	☐	Clinical data
☒	☐	Dual use research of concern

Methods

n/a	Involved in the study	
☒	☐	ChIP-seq
☒	☐	Flow cytometry
☒	☐	MRI-based neuroimaging

Antibodies

Antibodies used

Monoclonal anti-His Tag antibody produced in mouse, 1 mg/mL, clone 6G2AG, Protein A purified, lyophilized (ABIN387699) antibodies-online GmbH (Aachen, Germany); polyclonal anti-Mouse IgG-Atto 633 antibody produced in goat (78102- 6 1ML-F) Sigma Aldrich; polyclonal anti-mouse ADAMTS4 antibody produced in rabbit (ADAMTS4 Polyclonal Antibody, PA1-1749A, Invitrogen), Thermo Fisher Scientific (Waltham, Massachusetts, USA); monoclonal anti- mouse CD68 antibody produced in rat (MCA1957, clone FA-11), Bio-Rad (California, USA); polyclonal secondary anti-rabbit antibody AlexaFluor 647 produced in donkey (Donkey anti Rabbit IgG (H+L) Highly Cross-Adsorbed Secondary Antibody, Alexa Fluor Plus 647; Invitrogen, A32795), Thermo Fisher Scientific, Germany; anti-rat AlexaFluor 568, produced in goat (Goat anti-Tar IgG (H+L) Cross-Adsorbed Secondary Antibody, Alexa Fluor 568; Invitrogen,

	A-11077), Thermo Fisher Scientific, Germany; polyclonal anti-SMA-ab produced in rabbit (E-AB-34268.20), Elabscience, TX, USA; polyclonal secondary goat-anti-rabbit antibody Alexa Fluor 488 (ab10085), Abcam, Cambridge, UK; polyclonal anti-ADAMTS4-ab produced in rabbit (ab185722), Abcam, Cambridge, UK; polyclonal secondary donkey-anti-mouse ab Alexa Fluor 568 (A10042), Thermo Fisher Scientific, MA, USA; monoclonal anti-CD68-ab produced in rat (MCA1957GA), Biorad, Feldkirchen, Germany; polyclonal secondary goat-anti-rat-ab Alexa Fluor 568 (A11077), Thermo Fisher Scientific, MA, USA.; Goat anti-rabbit IgG:HRP (32460, Invitrogen); Rabbit anti-rat IgG:HRP (STAR21B, Bio-Rad)
Validation	The six used primary antibodies (anti-His Tag, anti-ADAMTS4, anti-SMA and anti-CD68) were validated by the supplier by using western blot staining and immunfluorescence. For the ADAMTS4 antibody, an advanced verification for binding the antigen was performed. All antibodies were already used and published in several references:<br>https://www.antikoerper-online.de/antibody/387699/anti-His+Tag+antibody/<br>https://www.thermofisher.com/antibody/product/ADAMTS4-Antibody-Polyclonal/PA1-1749A<br>https://www.biomol.com/de/produkte/antikoerper/primaerantikoerper/allgemein/anti-alpha-sma-e-ab-34268.20<br>https://www.abcam.com/adamts4-antibody-ab185722.html<br>https://www.bio-rad-antibodies.com/monoclonal/mouse-cd68-antibody-fa-11-mca1957.html?f=purified<br>https://www.bio-rad-antibodies.com/monoclonal/mouse-cd68-antibody-fa-11-mca1957.html?f=purified

Eukaryotic cell lines

Policy information about cell lines

Cell line source(s)	C57BL/6 mouse primary aortic endothelial Cells (Cellbiologics, USA),<br>human aortic endothelial cells (ATCC®, USA).
Authentication	Cell lines were authenticated by the respective supplier.Both cell lines were tested for expression of specific markers by specific staining, immunofluorescence staining or FACS.
Mycoplasma contamination	Both cell lines tested negative for mycoplasma, bacteria, yeast and fungi contamination.
Commonly misidentified lines<br>(See ICLAC register)	The study did not use misidentified cell lines.

Animals and other organisms

Policy information about studies involving animals; ARRIVE guidelines recommended for reporting animal research

Laboratory animals	Male 8-week-old apolipoprotein E knockout (ApoE-/-) (B6.129P2-ApoEtm1Unc/J) mice. Mice were obtained from the Research Institute of Experimental Medicine at the Charité Berlin and maintained under barrier conditions, including a. dark/light cycle of 12 hours, a ambient temperature of 20 °C and humidity of 45%.
Wild animals	The study did not involve wild animals.
Field-collected samples	The study did not involve samples collected in the field.
Ethics oversight	All procedures and animal maintenance were performed according to the guidelines and regulations of the Federation of Laboratory Animal Science Associations (FELASA) and the local Guidelines and Provisions for Implementation of the Animal Welfare Act. All animal studies were apporved by the local authorities (Lageso Berlin)

Note that full information on the approval of the study protocol must also be provided in the manuscript.

Human research participants

Policy information about studies involving human research participants

Population characteristics	samples of ruptured human aortic aneurysm
Recruitment	Samples were provided by the biobank of the Department of Vascular Surgery of the MRI/TU Munich.
Ethics oversight	The ethics committee of the MRI/TU Munich approved the use of the samples.

Note that full information on the approval of the study protocol must also be provided in the manuscript.
