# Supplementary material for: ADAMTS4-specific MR probe to assess aortic aneurysms in vivo using synthetic peptide libraries
Source: Nat Commun. 2022 May 23;13:2867. doi: 10.1038/s41467-022-30464-8 (PMC9126943; doi:10.1038/s41467-022-30464-8)
Supplement: Supplementary file 4 — Description of Additional Supplementary Files [file 41467_2022_30464_MOESM4_ESM.pdf]

Title: Supplementary Movie 1

Description: We performed a dynamic simulation, in which the peptide-protein complex was stable during the MD time of 0.1 µs. The MD simulation was performed at 310 K (37 °C) and a pressure of 1 bar.
